# Attention-Enhanced GAN for Astronomical Image Restoration Under Atmospheric Turbulence and Optical Aberrations

**DOI:** 10.3390/s26072135

**Published:** 2026-03-30

**Authors:** Chaoyong Peng, Jinlong Li, Jiaqi Bao, Lin Luo

**Affiliations:** School of Physical Science and Technology, Southwest Jiaotong University, Chengdu 611756, China; jinlong_lee@swjtu.edu.cn (J.L.); bjq838910432@163.com (J.B.); luolin@swjtu.edu.cn (L.L.)

**Keywords:** astronomical image restoration, atmospheric turbulence, deterministic optical aberrations, generative adversarial network, attention mechanism

## Abstract

Ground-based astronomical images are often degraded by atmospheric turbulence and deterministic optical aberrations introduced by telescope design and manufacturing processes. Joint mitigation of these distortions remains challenging due to the lack of reliable ground-truth data. To address this issue, a physics-based atmospheric–optical imaging model is developed to generate a large-scale, physically consistent simulated dataset, enabling supervised learning without real paired observations. Based on this, an attention-enhanced generative adversarial network (AE-GAN) is proposed for astronomical image restoration. The network incorporates a Channel Attention Block (CAB) and a Semantic Attention Module (SAM) within a feature pyramid architecture to enhance multi-scale representation and suppress turbulence-induced distortions. Experimental results show that the proposed method achieves consistent restoration performance under varying turbulence strengths, aberration amplitudes, and noise levels. Compared with recent Transformer-based methods, it maintains competitive performance across different aberration types while achieving significantly higher computational efficiency (1.21 s per image, 3.5× faster). In addition, the model trained on simulated data generalizes effectively to real astronomical observations.

## 1. Introduction

Mitigating the impact of atmospheric turbulence remains a fundamental challenge in image restoration research [1,2,3]. For ground-based astronomical observations, atmospheric turbulence severely degrades image resolution, while deterministic optical aberrations introduced by telescope design and manufacturing processes further deteriorate image quality [4]. Traditional model-based optimization methods have been widely used to improve imaging resolution; however, their reliance on iterative optimization often results in high computational cost and limited efficiency.

Deep learning (DL)-based approaches have demonstrated strong capability in image restoration with significantly improved computational efficiency, making them promising for astronomical imaging. However, imaging through atmospheric turbulence and optical aberrations is inherently complex. The point spread function (PSF) is stochastic, time-varying, and spatially varying, and turbulence can introduce not only blur but also complex distortions [5]. These factors make it difficult to obtain reliable ground-truth data and construct physically meaningful datasets for supervised learning in astronomical imaging scenarios.

From a physical perspective, atmospheric turbulence arises from random fluctuations in the refractive index across both spatial and temporal domains. Fried [6] introduced the atmospheric coherence length $r_{0}$ to quantify turbulence strength, which remains a fundamental parameter in modern estimation approaches [7,8]. The resulting wavefront phase distortions are commonly described using statistical turbulence models and can be simulated through random phase screen methods [9], forming the basis for numerical modeling of turbulence-induced imaging degradation. Recent studies further integrate these models into adaptive optics and simulation frameworks for real-time wavefront reconstruction and correction [10].

In practical imaging systems, image quality is further affected by deterministic optical aberrations, sensor noise, and the intrinsically low brightness of astronomical targets. As a result, the observed image is typically modeled as a convolution process governed by a joint atmospheric–optical PSF. However, accurately modeling and inverting this joint degradation remains challenging in practice.

A wide range of astronomical image restoration methods has been developed, including speckle imaging, blind deconvolution, adaptive optics, and DL-based post-processing techniques. However, most existing approaches primarily focus on atmospheric turbulence alone, while deterministic optical aberrations are often neglected or only implicitly considered. In addition, many learning-based methods rely on simplified turbulence simulations or natural-image datasets, leading to limited generalization under realistic astronomical imaging conditions. Related efforts have explored integrating physical simulators into DL frameworks to improve generalization under turbulence-induced distortions [11]. However, these methods are typically limited to turbulence-only scenarios and do not explicitly address joint turbulence–aberration effects.

To address these limitations, we propose a framework that combines physics-based modeling with learning-based restoration. Specifically, a physics-based atmospheric–optical imaging model is developed to simulate realistic degradation processes and generate a large-scale dataset for supervised training. Based on this, a GAN-based restoration framework is proposed.

The novelty of this work lies in the task-specific integration of a physics-based imaging model with an attention-enhanced restoration network to jointly address data scarcity and the complexity of turbulence–aberration degradation in astronomical imaging. The main contributions of this work can be summarized as follows:

1. A physics-based imaging model for astronomical image degradation. A model is developed that explicitly considers deterministic optical aberrations introduced by telescope design and manufacturing processes together with atmospheric turbulence, enabling realistic simulation of ground-based astronomical imaging;
2. A simulated dataset for turbulence- and aberration-degraded astronomical images. Based on the proposed imaging model and telescope parameters, a simulated dataset is generated to reproduce realistic imaging conditions and provide training data when large-scale real datasets are unavailable;
3. An attention-enhanced GAN (AE-GAN) framework for astronomical image restoration. An AE-GAN is designed to restore images degraded by turbulence and deterministic optical aberrations. The generator adopts a feature pyramid architecture with a Channel Attention Block (CAB) for channel recalibration and a Semantic Attention Module (SAM) for semantic-guided spatial refinement, improving the recovery of weak astronomical structures.

The remainder of this work is organized as follows: Section 2 reviews related work. Section 3 details the proposed imaging model and network architecture. Section 4 presents the dataset, evaluation metrics, and experimental results. Section 5 concludes the paper.

## 2. Related Work

High-resolution imaging of celestial objects through atmospheric turbulence has been extensively studied. Since Labeyrie’s pioneering work on speckle interferometry [12], this technique remains widely adopted in modern astronomical observations for high-angular-resolution imaging [13,14]. Speckle imaging exploits short-exposure frames to mitigate turbulence effects and achieve near diffraction-limited resolution. More recently, advanced reconstruction strategies have been integrated with speckle imaging. For example, Howell et al. [15] demonstrated that combining speckle observations with multi-frame blind deconvolution enables improved image restoration and enhances the recovery of fine structural details.

Blind deconvolution approaches aim to reconstruct the underlying object from short-exposure images without reference star information by jointly estimating the object and the unknown PSF. Early methods relied on iterative optimization frameworks, such as optical-flow-based and maximum-likelihood formulations [16,17,18] but often suffered from high computational cost and sensitivity to noise [19]. More recent studies have improved both modeling accuracy and computational efficiency. For example, Fétick et al. [20] proposed a parametric marginal approach within adaptive optics, while Ramos et al. [21] introduced a deep learning–accelerated multi-frame blind deconvolution method, thereby reducing computational complexity.

With the rapid development of DL, a wide range of learning-based approaches for image restoration have emerged. Many researchers have adopted U-Net–type architectures for image restoration tasks [22]. Sun et al. [23] designed a convolutional neural network (CNN) to predict motion blur and incorporated image priors for non-uniform deblurring. Liu et al. [24] proposed a two-stage deblurring framework based on high-frequency residual learning to better recover fine image details. In the first stage, an estimated blur kernel is used for deblurring, while in the second stage an encoder–decoder network refines the residual image.

More recently, deep learning approaches have been applied to atmospheric turbulence mitigation. Gao et al. [25] introduced a self-encoding deep CNN for restoring turbulence-degraded images using simulated atmospheric PSFs; however, the employed PSF model is simplified and fixed. Zhai et al. [26] developed a CNN model for turbulent distortion and aberration correction, enabling rapid compensation for turbulence-induced intensity fluctuations through supervised learning. Guo and Yang et al. [27,28] treated atmospheric turbulence as a form of random perturbation aberration and designed restoration networks inspired by noise-processing strategies. They further adopted a curriculum learning scheme that gradually increases degradation complexity. GAN-based approaches have also been explored for atmospheric turbulence mitigation, including recurrent GAN frameworks for video restoration that model spatial and temporal dependencies under turbulence distortion [29]. Adaptive optics (AO) systems are widely used to mitigate atmospheric turbulence. However, AO performance is fundamentally limited by wavefront sensor accuracy, which often leaves residual aberrations in the corrected images. These residual distortions can be further compensated by using deep convolutional neural networks (CNNs) in post-processing frameworks [30,31,32].

Despite the progress of DL-based restoration methods, several challenges remain. First, many approaches rely on datasets constructed from natural images with simplified turbulence simulations, which may not accurately reflect realistic astronomical imaging conditions. Second, astronomical images differ significantly from natural scenes, exhibiting extremely low brightness, sparse structures, and near-black backgrounds, which limits model generalization. Moreover, most existing methods focus on turbulence-only degradation, whereas real observations are often affected by both atmospheric turbulence and deterministic optical aberrations. Finally, the lack of widely accepted benchmarks and the mismatch between existing methods (e.g., terrestrial scenes or multi-frame restoration) and single-image astronomical settings further hinder fair comparison and practical applicability. These limitations highlight the need for restoration frameworks that explicitly model joint turbulence–aberration degradation under realistic astronomical conditions, which motivates the approach proposed in this work.

## 3. Method

In recent years, generative adversarial networks (GANs) have shown strong capability in image restoration tasks and have therefore attracted increasing attention in astronomical imaging. In this work, we investigate the feasibility of using a GAN-based framework for restoring astronomical images degraded by atmospheric turbulence and deterministic optical aberrations. Because sufficient real aberration-degraded astronomical data are difficult to acquire, we further develop a physics-based imaging model to generate a simulated dataset for training and evaluation.

### 3.1. Atmospheric–Optical Aberration Imaging

The following imaging model is constructed by integrating established formulations from optical imaging theory, primary aberration modeling, and atmospheric turbulence phase-screen modeling to simulate joint degradation in ground-based astronomical imaging.

For ground-based astronomical imaging, the atmosphere and telescope can be modeled jointly as an integrated imaging system. Under this assumption, the observed image is expressed as the convolution of the object with the point spread function (PSF):

(1) $$i\left(x,y\right)=h\left(x,y\right)*o\left(x,y\right)$$

where $\left(x,y\right)$ represents the 2D coordinate, ∗ represents convolution, $i\left(x,y\right)$ is the observed image through the atmosphere, $o\left(x,y\right)$ is the ideal object image, and $h\left(x,y\right)$ is the PSF of the atmospheric–optical system. Noise and atmospheric absorption are not considered in the above imaging model formulation. The effect of sensor noise is incorporated later during dataset generation. In the presence of deterministic optical aberrations, the PSF is given by

(2) $$h(x,y)=|\iint_{S_{D}}P_{0}\left(\xi ,\eta \right)\;exp\;\{j[\Delta \varphi_{S}\left(\xi ,\eta \right)+\Delta \varphi_{D}\left(\xi ,\eta \right)]\}\;exp\;[j2\pi (\xi x+\eta y)]d\xi d\eta {|}^{2}$$

where $\left(\xi ,\eta \right)$ denotes normalized spatial frequency coordinates in the pupil plane; $P_{0}\left(\xi ,\eta \right)$ is the pupil function; $\Delta \varphi_{S}\left(\xi ,\eta \right)$ and $\Delta \varphi_{D}\left(\xi ,\eta \right)$ represent the atmospheric random phase and the optical aberration-induced phase, respectively; and the corresponding wavefront aberrations are $\Delta W_{S}\left(\xi ,\eta \right)$ and $\Delta W_{D}\left(\xi ,\eta \right)$. Equation (2) indicates that the PSF of the atmospheric–optical system is jointly determined by atmospheric random phase distortions and deterministic optical aberrations.

The wavefront aberration of the primary optical system is approximated using low-order polynomial terms corresponding to defocus, spherical aberration, coma, and astigmatism, and can be expressed as [33]

(3) $$W_{D}(\xi ,\eta )=A(\xi^{2}+\eta^{2})+B(\xi^{2}+\eta^{2}{)}^{2}+C\eta (\xi^{2}+\eta^{2})+D(\xi^{2}+3\eta^{2})$$

where A, B, C and D correspond to the defocus, spherical aberration, coma, and astigmatism coefficients, respectively.

To model atmospheric turbulence, a random phase screen is introduced at the entrance pupil, and the corresponding random phase under simplified assumptions can be written as

(4) $$\Delta \varphi_{S}(\xi ,\eta )=C\iint_{\infty }R(\overset{⃑}{k})\sqrt{F_{\varphi }(\vec{k})}e^{j\vec{u}\cdot \overset{⃑}{k}}d\overset{⃑}{k}$$

where $\vec{u}=\left(\xi ,\eta \right)$ is a 2D coordinate vector, $R(\overset{⃑}{k})$ is a Gaussian random process, $\overset{⃑}{k}$ is the spatial wavenumber vector, C is a constant that scales the random phase according to the turbulence intensity, and $F_{\phi }(\overset{⃑}{k})$ is the Kolmogorov-spectrum-based phase fluctuation power spectrum [34]:

(5) $$F_{\phi }\left(\overset{⃑}{k}\right)=0.490{r_{0}}^{-5/3}{\overset{⃑}{k}}^{-11/6}$$

where $r_{0}$ is the atmospheric coherence length, indicating the strength of atmospheric turbulence.

The corresponding wavefront aberration is

(6) $$\Delta W\left(\xi ,\eta \right)=\frac{\lambda }{2\pi }\Delta \varphi \left(\xi ,\eta \right)$$

The atmospheric random phase can be converted into the corresponding wavefront aberration, and the total wavefront aberration is obtained by combining the deterministic system aberration with the turbulence-induced disturbance. Thus, the total wavefront aberration at the pupil is the sum of the system aberration and the atmospheric disturbance:

(7) $$\Delta W\left(\xi ,\eta \right)=\Delta W_{D}\left(\xi ,\eta \right)+\Delta W_{S}\left(\xi ,\eta \right)$$

Using the above imaging model, we generate a simulated astronomical image dataset incorporating both atmospheric turbulence and optical system aberrations.

### 3.2. Network Architecture

**Figure 1:**
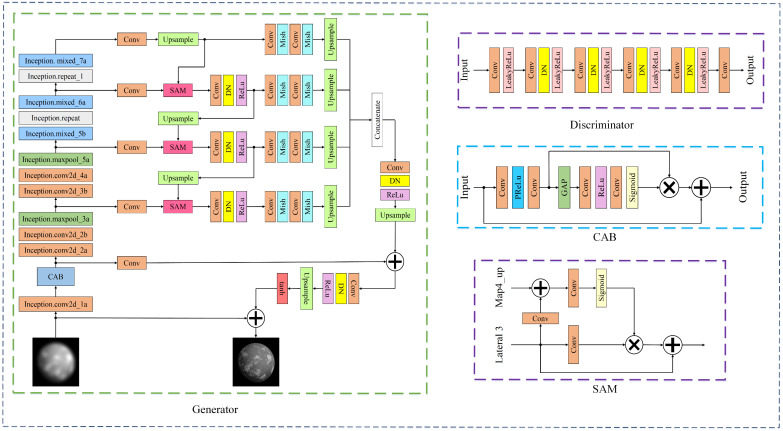
Architecture of the proposed network. The network consists of two main components: a generator that produces restored images from blurred inputs, and a discriminator that distinguishes between generated images and ground-truth images.

To restore high-quality astronomical images from degraded observations, we design a GAN-based framework. The network consists of two main components, namely a generator G, which produces restored images, and a discriminator D, which distinguishes generated images from target images, as illustrated in Figure 1. Within this architecture, CAB is applied at the shallow feature stage, while SAM is embedded within the feature pyramid to guide cross-scale feature fusion.

#### 3.2.1. Generator Design

To enable the discriminator to better distinguish generated images from target samples, we define the loss function $L_{D}$ as follows:

(8) $$L_{D}=E_{I_{B}\sim p_{data}(I_{B})}\left[{\left(D(I_{B})-E_{n\sim p_{n}(n)}D(G(n))-1\right)}^{2}\right]+E_{n\sim p_{n}(n)}\left[{\left(D(G(n))-E_{I_{B}\sim p_{data}(I_{B})}D(I_{B})+1\right)}^{2}\right]$$

where $D\left(\cdot \right)$ denotes the discriminator output, G(n) is the generated result by the generator, $I_{B}$ represents the blurred input image, and  n is the random noise vector. This design improves training stability and helps enhance the perceptual quality of restored images [35]. In addition, a PatchGAN discriminator [36] is adopted to emphasize local texture consistency. To better handle severely blurred images and complex structures, a global-local discrimination strategy is further used so that both global contextual information and local details can be exploited during training.

The generator architecture is inspired by feature pyramid structures (FPNs) [37], which have proven effective in capturing multi-scale and multi-level semantic features. To this end, we extract feature maps of different spatial resolutions from each layer of the backbone network and organize them into a feature pyramid. The generator adopts an Inception-ResNet backbone [38] for feature extraction because it provides strong multi-scale representation capability and stable optimization through deep residual connections. This property is beneficial for astronomical images containing structures at multiple spatial scales.

To improve training stability when real astronomical data are limited, the backbone is initialized using ImageNet pre-training [39], which provides generic low-level visual priors such as edges and texture patterns. During training, the simulation pipeline performs physics-based domain randomization by sampling a wide range of turbulence strengths, optical aberration coefficients (e.g., defocus, coma, and astigmatism), and noise levels. This exposes the network to diverse degradation patterns during training and improves generalization beyond a single fixed distribution.

Feature maps extracted from different backbone stages are organized into a feature pyramid structure. Multi-scale feature maps are progressively upsampled and fused so that the generator can simultaneously utilize high-level semantic information and low-level spatial details. Each feature level is processed using a feature pyramid head consisting of convolutional layers, denoising (DN) operations, and nonlinear activations (ReLU and Mish). The resulting feature maps are upsampled to a common spatial resolution and concatenated along the channel dimension. The fused representation is further refined by convolutional layers and merged with the shallow feature map ($map_{0}$) to preserve fine spatial details.

#### 3.2.2. Attention Mechanisms

1. Channel Attention Block

To enhance feature discrimination at the shallow stage, a Channel Attention Block (CAB) is introduced to adaptively recalibrate channel-wise feature responses. Given an input feature map M, several convolutional layers are first applied for local feature refinement, followed by global average pooling to generate a 1 × 1 × n channel descriptor. This descriptor is then transformed through two convolutional layers and a sigmoid activation to produce channel-wise weights $w^{\prime }$ in the range [0, 1]. The weights are used to rescale the channels of M, yielding a recalibrated feature map that is finally combined with the original input through a residual connection.

This mechanism enables the network to automatically learn the relative importance of different feature channels. By suppressing turbulence-dominated or less informative responses and emphasizing reliable structural cues, CAB stabilizes shallow feature representations before multi-scale feature fusion. Although CAB shares the general idea of channel reweighting with standard channel attention modules such as SE or ECA, it is specifically designed for turbulence- and aberration-degraded astronomical restoration. By performing lightweight local refinement prior to channel gating and applying residual recalibration, CAB helps mitigate the influence of stochastic turbulence-induced blur and speckle patterns in early feature representations.

2. Semantic Attention Module

To further improve cross-scale feature consistency, a Semantic Attention Module (SAM) is incorporated within the feature pyramid. The SAM utilizes high-level feature maps as semantic guidance to refine lower-level feature maps through adaptive weighted correction. Specifically, semantic features are used to generate an attention map that modulates the corresponding low-level features, and the corrected representation is added to the original feature map to produce the output.

Unlike standard skip connections or simple pyramid aggregation schemes (e.g., U-Net++ or conventional FPNs), SAM introduces semantic priors from high-level representations to guide low-level feature refinement. This design is particularly beneficial for astronomical image restoration, where deterministic optical aberrations introduce spatially structured distortions. By injecting semantic guidance during multi-scale feature fusion, SAM improves the recovery of fine structures while maintaining cross-scale consistency under combined turbulence and deterministic optical aberrations.

The proposed framework targets a joint degradation scenario in which stochastic turbulence-induced distortions and deterministic optical aberrations coexist. Within the feature pyramid hierarchy, CAB suppresses turbulence-dominated channel responses and enhances reliable structural features, while SAM provides semantic guidance to stabilize the recovery of fine details. Together, these modules improve the robustness of the network when restoring astronomical images affected by coupled turbulence and deterministic optical aberrations.

### 3.3. Loss Function

To achieve high-quality image restoration, the generator is trained using a composite loss that integrates three complementary components. The overall generator loss is defined as

(9) $$L_{G}=\lambda_{1}L_{MSE}+\lambda_{2}L_{PL}+\lambda_{3}L_{adv}$$

where $\lambda_{1}$, $\lambda_{2}$ and $\lambda_{3}$ are empirically determined weighting coefficients, set to $\lambda_{1}=0.5$, $\lambda_{2}=0.006$ and $\lambda_{3}=0.001$.

The mean squared error (MSE) loss $L_{MSE}$ enforces pixel-wise similarity between the restored image and the ground-truth sharp image, thereby constraining low-level reconstruction errors. It is defined as

(10) $$L_{MSE}=\frac{1}{r^{2}WH}\sum_{x=1}^{rW}\sum_{y=1}^{rH}{\left({\left(I_{S}\right)}_{x,y}-G_{\theta_{G}}{\left(I_{B}\right)}_{x,y}\right)}^{2}$$

where r denotes the scaling factor, W and H represent the width and height of the input image, and $I_{S}$ denotes the ground-truth sharp image.

The perceptual loss $L_{PL}$ [40] measures the difference between high-level feature representations extracted from the ground-truth and generated images. By comparing feature activations in a pretrained VGG19 network, the perceptual loss encourages the restored image to preserve global structure and semantic content. It is formulated as

(11) $$L_{PL}=\frac{1}{W_{i,j}H_{i,j}}\sum_{x=1}^{W_{i,j}}\sum_{y=1}^{H_{i,j}}{\left(\varphi_{i,j}(I_{S}{)}_{x,y}-\varphi_{i,j}{\left(G_{\theta_{G}}\left(I_{B}\right)\right)}_{x,y}\right)}^{2}$$

where $\varphi_{i,j}$ denotes the feature map extracted from the j-th convolution layer before the i-th max-pooling layer within the VGG19 network pretrained on ImageNet, and $W_{i,j}$ and $H_{i,j}$ represent the dimensions of the corresponding feature map.

The adversarial loss $L_{\mathrm{adv}}$ is defined using the Wasserstein GAN (WGAN) formulation [41], which improves training stability and encourages the generator to produce perceptually realistic textures. It is expressed as

(12) $$L_{adv}=\sum_{n=1}^{N}-D_{\theta_{D}}\left(G_{\theta_{G}}\left(I_{B}\right)\right)$$

where $D_{\theta_{D}}$ denotes the discriminator output.

The three loss terms play complementary roles in training. The MSE loss constrains pixel-level reconstruction, the perceptual loss promotes structural consistency and detail preservation, and the adversarial loss reduces over-smoothing while improving the perceptual realism of restored textures. The loss weights were selected following common practice in GAN-based restoration and further adjusted through empirical tuning on a validation subset to balance numerical fidelity and perceptual sharpness. The final hyperparameter settings are reported to facilitate reproducibility. For clarity, the overall training and inference procedure of the proposed AE-GAN framework is summarized in Algorithm 1, while the exact loss definitions used in optimization are given in Equations (8)–(12).
**Algorithm 1:**  Training and inference procedure of the proposed AE-GAN**Input**: blurred image *y*, ground truth image *x* (simulated training only); generator G(.); discriminator D(.); Loss weights λ_1_, λ_2_, λ_3_ **Output**: restored image $\hat{x}$ **1**: **Initialize** generator parameters *θ_G_* and discriminator parameters *θ_D_* 2: **for** each training iteration **do** **3**: Sample a mini-batch of paired data $\{(x_{i},y_{i}){\}}_{i=1}^{B}$ **4**: **Generator forward:**
${\hat{x}}_{i}=y_{i}+G(y_{i})$ 5: **Update discriminator:** 6:       ${\;L}_{D}=E[D\left(\hat{x}\right)]-E[D(x)]$ 7:        $\theta_{D}\leftarrow \theta_{D}-\eta \nabla_{\theta_{D}}L_{D}$ 8: **Compute generator losses:** 9:         $L_{MSE}={\left\|\hat{x}-x\right\|}_{2}^{2}$ 10:       $L_{PL}={\left\|\phi \left(\hat{x}\right)-\phi \left(x\right)\right\|}_{2}^{2}$ 11:       $L_{adv}=-E[D\left(\hat{x}\right)]$ 12: **Update generator:** 13:       $\;L_{G}=\lambda_{1}L_{MSE}+\lambda_{2}L_{PL}+\lambda_{3}L_{adv}$ 14:       $\;\theta_{G}\leftarrow \theta_{G}-\eta \nabla_{\theta_{G}}L_{G}$ 15: **end for** 16: **Inference:** given a real blurred observation *y*, output $\hat{x}=y+G(y)$


## 4. Experiments

### 4.1. Implementation Details

All experiments were conducted using the PyTorch (version 2.2) framework. Training and testing were performed on a Windows 10 workstation equipped with an Intel i7-7700 CPU (3.6 GHz) and an NVIDIA GTX 1080 Ti GPU. The dataset used for training and testing was generated using the imaging model introduced in Section 3.1. The network was trained for 1500 epochs using the Adam optimizer.

During training, the initial learning rate was set to $10^{-4}$ and then linearly decayed at a rate of $10^{-7}$. To quantitatively evaluate the restoration performance, two commonly used image quality metrics were adopted: the peak signal-to-noise ratio (PSNR) and the structural similarity index (SSIM). The generator contains approximately 21.75 M parameters and requires 57.10 G FLOPs. Most of the computational cost originates from the Inception-based backbone and the multi-scale branches, while the attention modules (CAB and SAM) are lightweight and introduce only marginal overhead.

### 4.2. Dataset Generation

The astronomical image dataset used in this work was generated based on the physics-based atmospheric–optical imaging model described in Section 3.1, following the general simulation framework proposed in Ref. [42]. Compared with that framework, the present study further incorporates time-dependent atmospheric turbulence, optical system aberrations, and sensor noise. The main parameters of the simulated telescope imaging system are summarized in Table 1.

Ground-truth images were selected from Solar System targets available in the NASA Image Library. For each celestial object, three imaging conditions were simulated:

1. Atmospheric turbulence only;
2. Atmospheric turbulence combined with deterministic optical aberrations;
3. Atmospheric turbulence with additive noise.

The strength of atmospheric turbulence was characterized by the parameter $D/r_{0}$, which was randomly sampled in the range of 1–30. Deterministic optical aberrations were simulated with amplitudes randomly selected between 0.5λ and 3λ, including defocus, spherical aberration, coma, and astigmatism. Gaussian noise was added with signal-to-noise ratios ranging from 5 dB to 50 dB.

**Figure 2:**
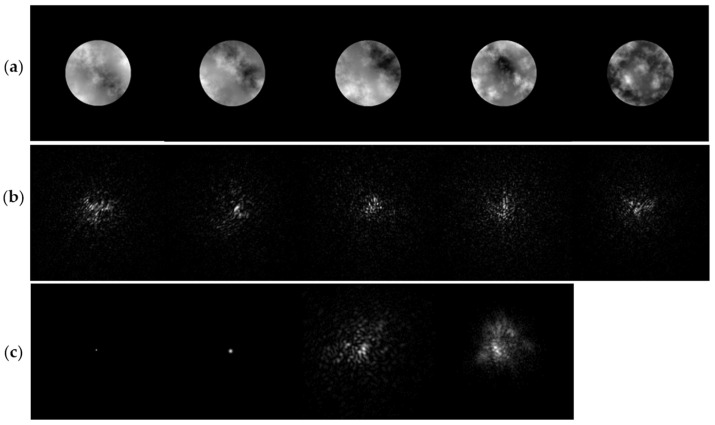
Simulated random phase screens and PSF images: (**a**) Random phase screens; (**b**) corresponding PSF images; (**c**) shows single-star images from left to right: original diffraction-limited image, simulated diffraction image, simulated speckle image, and real Orion-σ star image.

Figure 2 presents examples of simulated random phase screens and the corresponding PSFs. The phase screens and PSFs were generated according to the Kolmogorov statistical model with $D/r_{0}\;=\;10$. In Figure 2c, simulated single-star images are compared with real telescope observations of the Orion-σ star. The comparison shows that the simulated turbulence-induced speckle patterns closely resemble those observed in real astronomical observations, which validates the physical fidelity of the simulation model.

**Figure 3:**
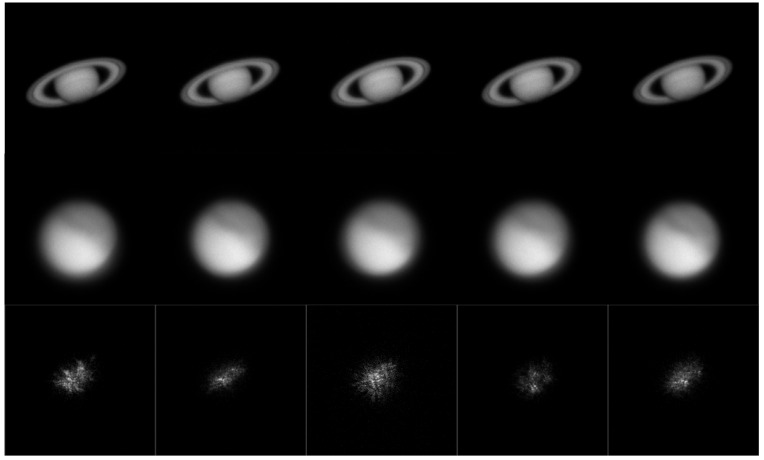
Examples of real astronomical images captured at Yunnan Astronomical Observatory.

To further evaluate the performance of the proposed framework on real astronomical observations, images of Saturn, Mars, and Orion-σ were collected using the optical telescope at Yunnan Astronomical Observatory, China (Figure 3). The data were captured under strong turbulence conditions. Under such conditions, the recorded images often exhibit shaking, deformation, and occasional defocus due to difficulties in accurately maintaining the target within the field of view.

**Figure 4:**
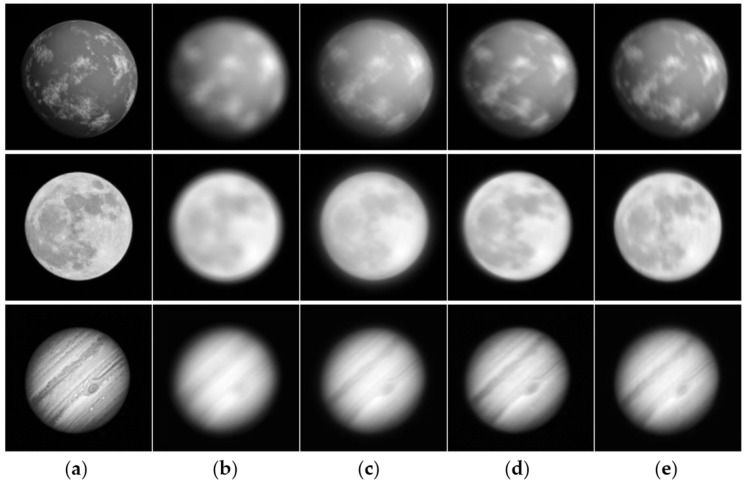
Example simulated images under $D/r_{0}\;=\;10$: (**a**) ground truth; (**b**) defocus; (**c**) spherical aberration; (**d**) coma; (**e**) astigmatism.

A total of 36,000 simulated images with a resolution of 512 × 512 were generated. Examples of the simulated data under $D/r_{0}\;=\;10$ with four types of deterministic optical aberrations are shown in Figure 4. Among all samples, 30,000 images were used for training and 6000 for testing. The detailed dataset configuration is summarized in Table 2.

### 4.3. Ablation Study

To quantify the effectiveness of the proposed CAB and SAM, we conduct ablation experiments by selectively disabling these components. All models were trained under the same conditions ($D/r_{0}\;=\;10$, optical aberration amplitude of 1.5λ) using the Inception-ResNet backbone. The quantitative results are summarized in Table 3.

As shown in Table 3, the baseline configuration (Index 1) only yields a PSNR of 24.51 dB. The addition of CAB (Index 2) results in a 1.30 dB improvement, indicating its effectiveness in recalibrating channel responses under turbulence-induced random blur. When SAM is integrated (Index 3), the SSIM improves significantly by 0.10, indicating its effectiveness in recovering fine-grained details using high-level semantic priors. The full configuration (Index 4) achieves an overall gain of 3.89 dB in PSNR and 0.13 in SSIM compared to the backbone, supporting the effectiveness of the proposed architectural design for astronomical image restoration.

### 4.4. Comparison with Existing Methods

To demonstrate the effectiveness of the proposed framework, we compare it against four representative image restoration algorithms, including one classical deconvolution method and three learning-based methods. Specifically, we select the classical Richardson–Lucy algorithm (L-R) [43], the method proposed by Tao et al. [44], the pioneering GAN-based DeblurGAN [45], and the recent state-of-the-art Transformer-based model TMT [46]. All learning-based models were retrained on the same physics-based astronomical dataset to ensure a fair comparison. While L-R remains a classical benchmark in astronomical imaging due to its rigorous statistical basis, its high computational cost and sensitivity to noise limit its applicability in real-time high-resolution observations. Our method retains the physical motivation of traditional restoration approaches while leveraging deep learning to avoid iterative optimization bottlenecks.

**Figure 5:**
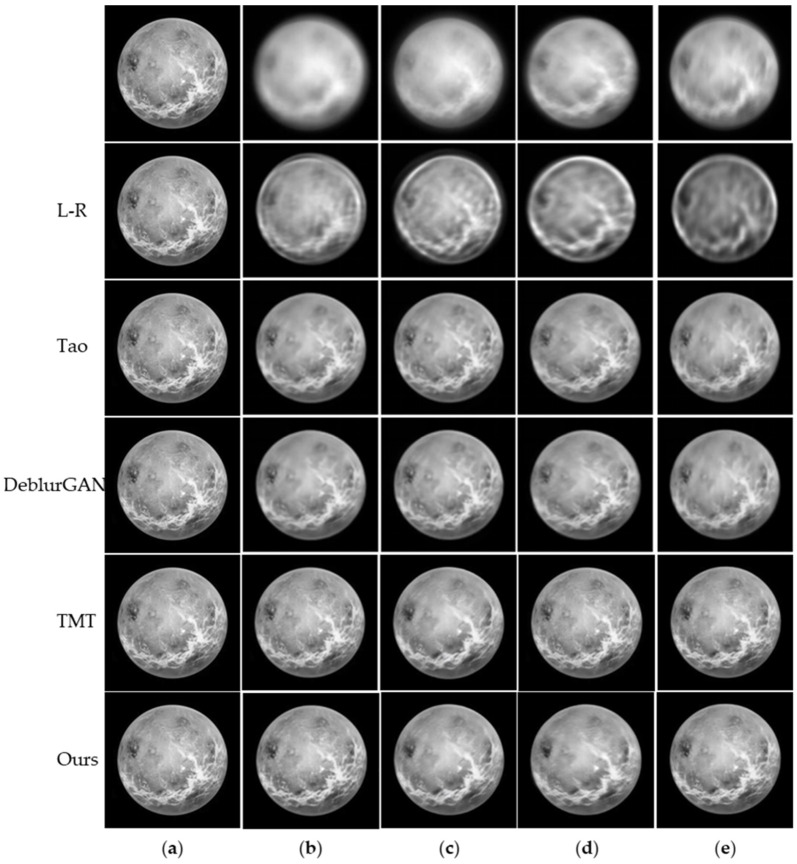
Restoration results of different methods: (**a**) Ground truth; (**b**) defocus; (**c**) spherical aberration; (**d**) coma; (**e**) astigmatism.

Qualitative restoration results under different aberration types are presented in Figure 5, where the proposed method exhibits clearer structural reconstruction and better preservation of fine details than the competing approaches. All methods were evaluated on simulated images with atmospheric turbulence ($D/r_{0}=10$) and an optical system aberration with an amplitude of 1.5λ. The output of the L-R method is obtained by directly applying L-R deconvolution to our simulated blurred images, while the results of Tao et al., DeblurGAN, and TMT are obtained using models retrained on the same dataset following their original implementation settings.

Quantitative comparisons using the PSNR and the SSIM are summarized in Table 4. The results demonstrate that the proposed AE-GAN consistently outperforms traditional and early deep-learning baselines (L-R, Tao et al., and DeblurGAN) across all aberration types, while achieving performance comparable to TMT.

For structured aberrations (spherical and astigmatism), AE-GAN achieves the best performance among all compared methods and further outperforms TMT. Specifically, AE-GAN improves PSNR by 0.16 dB over TMT for spherical aberration (28.47 vs. 28.31 dB) and by 0.21 dB for astigmatism (28.76 vs. 28.55 dB), indicating its effectiveness in handling structured wavefront distortions.

For spatially varying aberrations (defocus and coma), AE-GAN still demonstrates clear improvements over L-R, Tao et al., and DeblurGAN, while remaining close to TMT. In particular, the PSNR is 0.05 dB lower than that of TMT for defocus (28.40 vs. 28.45 dB) and 0.02 dB lower for coma (28.10 vs. 28.12 dB), indicating only marginal performance differences under these conditions. In terms of SSIM, TMT achieves slightly higher values (by up to 0.02–0.03) in some cases, likely due to its global self-attention mechanism. However, this advantage is relatively limited and does not translate into consistent gains in PSNR.

Notably, the proposed framework exhibits a clear advantage in computational efficiency. The inference time for a single image is 4.18 s for TMT, whereas AE-GAN requires only 1.21 s, achieving a 3.5× speedup.

Overall, the proposed AE-GAN provides a favorable balance between restoration performance and computational efficiency, making it well suited for practical astronomical imaging applications. These results suggest that the proposed framework is particularly effective for structured aberrations, while remaining competitive under spatially varying aberrations.

### 4.5. Results Under Different Atmospheric Turbulence Strengths

To evaluate the robustness of the proposed framework under different atmospheric conditions, experiments were conducted on simulated images without deterministic optical aberrations at five turbulence levels, $D/r_{0}\;=\;25,20,15,10,5$. For each turbulence level, representative images were selected from the test dataset. A larger $D/r_{0}$ corresponds to stronger atmospheric turbulence, whereas a smaller value indicates more favorable imaging conditions. The results are summarized in Table 5.

The restoration performance monotonically decreases with increasing turbulence strength; however, the proposed framework still maintains competitive performance even under strong turbulence.

### 4.6. Results Under Varying Aberration Amplitudes

To further assess the performance of the proposed framework under different optical aberration strengths, four primary aberration types—defocus, spherical aberration, coma, and astigmatism—were evaluated at three aberration magnitudes: λ, 2λ, and 3λ. All experiments were conducted under atmospheric turbulence with $D/r_{0}\;=\;10$. Experiments involving mixed aberrations were also included, where the four aberration types were randomly combined and their amplitudes were uniformly sampled within the same λ, 2λ, and 3λ ranges. Since a large number of test images were evaluated, the results are summarized in Table 6 using PSNR and SSIM.

As expected, larger aberration amplitudes generally lead to reduced restoration performance due to the increased distortion introduced into the imaging process, and this trend is consistent across all aberration types. Nevertheless, even at an aberration amplitude of 3λ, the proposed framework maintains competitive reconstruction accuracy, demonstrating its robustness under severe deterministic optical aberrations.

### 4.7. Results on Noise-Degraded Images

**Figure 6:**
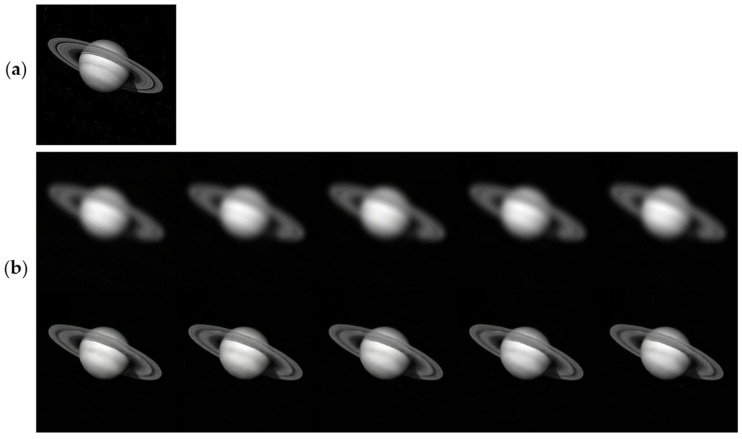
Restoration results for simulated noisy Saturn images. (**a**) Ground truth. (**b**) Top row: noisy inputs with SNR values of 20 dB, 25 dB, 30 dB, 35 dB, and 40 dB (from left to right); bottom row: corresponding restored results.

To further evaluate the robustness of the proposed framework to sensor noise, we selected simulated Saturn images with atmospheric turbulence of $D/r_{0}\;=\;10$ and a fixed defocus aberration of 2λ. Gaussian noise of different levels was added to generate noisy observations with SNR values of 20 dB, 25 dB, 30 dB, 35 dB, and 40 dB. Representative restoration results are shown in Figure 6, and the corresponding quantitative metrics are summarized in Table 7.

The PSNR and SSIM values remain consistently stable across all noise levels, with the PSNR ranging from 26.60 dB to 27.01 dB and the SSIM ranging from 0.80 to 0.83, indicating robust performance to varying noise levels. These results indicate that the proposed AE-GAN maintains stable restoration performance under different noise levels and is suitable for restoring turbulence-degraded astronomical images.

### 4.8. Results on Actual Astronomical Images

**Figure 7:**
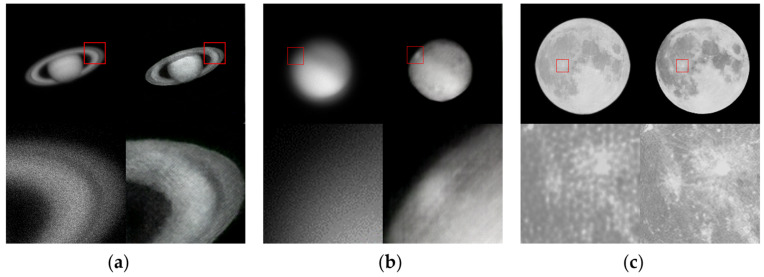
Experimental results on real astronomical image restoration. For each object, the original observation (top left), the restored image (top right), and the corresponding locally magnified regions (bottom) are shown. (**a**) Saturn. (**b**) Mars. (**c**) Moon. Red boxes indicate representative regions of interest (ROIs) selected for detailed visual comparison, highlighting enhanced structural reconstruction and improved preservation of local textures.

To evaluate the practical applicability of the proposed framework, real astronomical images were tested, including images of Saturn and Mars captured by the optical telescope at Yunnan Astronomical Observatory, China, as well as lunar images acquired using professional cameras. To rigorously assess the robustness of the network, images affected by relatively severe atmospheric turbulence were deliberately selected. The raw images are severely degraded by atmospheric turbulence, resulting in significant loss of structural details and blurred target contours. The restoration results are shown in Figure 7. After restoration using the proposed network, the structural fidelity of the images is noticeably improved: Saturn’s rings become more discernible, the polar ice cap on Mars is partially recovered, and surface features of the Moon are more clearly revealed.

These results indicate that the network, trained solely on simulated data, generalizes effectively to real-world astronomical observations. It is worth noting that our model is trained purely on physics-based simulated data and is directly applied to real astronomical observations without any real-domain fine-tuning. This setting is inherently challenging due to the inevitable simulated-to-real domain gap. Therefore, the consistent restoration performance on real targets (e.g., Saturn, Mars, and Moon) provides encouraging qualitative evidence that the proposed framework and simulation pipeline learn a transferable restoration mapping. Nevertheless, extremely rare real-world degradations that are not fully covered by the simulation may still introduce artifacts, which we discuss as limitations and future improvement directions.

## 5. Conclusions

This study demonstrates that astronomical images jointly degraded by atmospheric turbulence and deterministic optical aberrations can be effectively restored using a physics-informed learning framework, even in the absence of reliable ground-truth data and under the computational constraints of traditional iterative methods.

By integrating physics-based data generation with an attention-enhanced generative adversarial network (AE-GAN), the proposed approach enables supervised learning without relying on real paired observations. The results indicate consistent restoration performance across varying turbulence strengths, aberration types, and noise levels, while maintaining a favourable balance between restoration quality and computational efficiency. Compared with representative methods, the proposed framework achieves competitive restoration performance with significantly reduced inference time (1.21 s per image, approximately 3.5× faster than a Transformer-based model), highlighting its potential for practical astronomical imaging applications. Furthermore, the ability of the model trained solely on simulated data to generalize to real astronomical observations suggests that physics-based data generation can provide an effective pathway for addressing data scarcity in this domain.

Nevertheless, the current simulation framework may not fully capture all real-world degradations, which can lead to residual artifacts in more complex observational conditions. Future work will focus on improving the fidelity of the physical modelling and extending the proposed framework to multi-frame restoration scenarios.

## Figures and Tables

**Table 1:** Parameters of the simulated telescope imaging system.

Parameters	Value
Aperture D (m)	1.06
CCD pixel size (μm)	6.4
Image size (px)	512 × 512
Center wavelength λ (nm)	700
$D/r_{0}$	1–30
Aberration amplitude	0.5λ–3λ
SNR (dB)	5–50

**Table 2:** Summary of the simulated dataset and categories.

Dataset	Types	Number
Training data	Turbulence-only images	15,000
	Turbulence + aberration images	10,000
	Turbulence + noise images	5000
Testing data	Turbulence-only images	3000
	Turbulence + aberration images	2000
	Turbulence + noise images	1000

**Table 3:** Ablation study of the proposed components on the simulated dataset.

Index	Backbone	CAB	SAM	PSNR (dB)	SSIM
1	√			24.51	0.71
2	√	√		25.81	0.80
3	√		√	26.12	0.81
4 (ours)	√	√	√	28.40	0.84

**Table 4:** Quantitative comparison of restoration methods under different aberrations.

Methods	L-R [43]		Tao [44]		DeblurGAN [45]		TMT [46]		Ours	
Aberration	PSNR	SSIM	PSNR	SSIM	PSNR	SSIM	PSNR	SSIM	PSNR	SSIM
Defocus	21.74	0.71	27.03	0.81	26.85	0.79	28.45	0.86	28.40	0.84
Spherical	22.49	0.68	28.35	0.81	27.10	0.80	28.31	0.83	28.47	0.85
Coma	17.60	0.68	27.77	0.82	26.50	0.78	28.12	0.86	28.10	0.85
Astigmatism	16.75	0.70	27.77	0.81	26.65	0.79	28.55	0.83	28.76	0.84

**Table 5:** Restoration results under different atmospheric turbulence strengths.

$D/r_{0}$	25	20	15	10	5
PSNR	29.37	29.41	30.27	30.62	30.89
SSIM	0.86	0.87	0.87	0.89	0.91

**Table 6:** Restoration results for different optical aberration amplitudes.

$\Delta W_{D}$	1λ		2λ		3λ	
Aberration	PSNR	SSIM	PSNR	SSIM	PSNR	SSIM
Defocus	29.78	0.86	28.25	0.82	27.09	0.80
Spherical aberration	29.77	0.87	28.92	0.86	27.48	0.84
Coma	29.85	0.86	28.69	0.84	27.93	0.83
Astigmatism	29.61	0.87	29.02	0.84	27.84	0.83
Mixed	29.45	0.85	28.82	0.82	27.74	0.81

**Table 7:** Evaluation of restoration results for noisy images.

SNR (dB)	20	25	30	35	40
PSNR	26.60	26.67	26.89	26.99	27.01
SSIM	0.80	0.82	0.82	0.83	0.83

## Data Availability

Due to project-related constraints, we are willing to provide partially simulated data and detailed implementation information upon reasonable request after publishing.
